# Structural variants and short tandem repeats impact gene expression and splicing in bovine testis tissue

**DOI:** 10.1093/genetics/iyad161

**Published:** 2023-09-01

**Authors:** Meenu Bhati, Xena Marie Mapel, Audald Lloret-Villas, Hubert Pausch

**Affiliations:** Animal Genomics, ETH Zurich, Universitaetstrasse 2, 8092, Zurich, Switzerland; Animal Genomics, ETH Zurich, Universitaetstrasse 2, 8092, Zurich, Switzerland; Animal Genomics, ETH Zurich, Universitaetstrasse 2, 8092, Zurich, Switzerland; Animal Genomics, ETH Zurich, Universitaetstrasse 2, 8092, Zurich, Switzerland

**Keywords:** structural variants, short tandem repeats, cattle, expression/splicing QTL, testis

## Abstract

Structural variants (SVs) and short tandem repeats (STRs) are significant sources of genetic variation. However, the impacts of these variants on gene regulation have not been investigated in cattle. Here, we genotyped and characterized 19,408 SVs and 374,821 STRs in 183 bovine genomes and investigated their impact on molecular phenotypes derived from testis transcriptomes. We found that 71% STRs were multiallelic. The vast majority (95%) of STRs and SVs were in intergenic and intronic regions. Only 37% SVs and 40% STRs were in high linkage disequilibrium (LD) (*R*^2^ > 0.8) with surrounding SNPs/insertions and deletions (Indels), indicating that SNP-based association testing and genomic prediction are blind to a nonnegligible portion of genetic variation. We showed that both SVs and STRs were more than 2-fold enriched among expression and splicing QTL (e/sQTL) relative to SNPs/Indels and were often associated with differential expression and splicing of multiple genes. Deletions and duplications had larger impacts on splicing and expression than any other type of SV. Exonic duplications predominantly increased gene expression either through alternative splicing or other mechanisms, whereas expression- and splicing-associated STRs primarily resided in intronic regions and exhibited bimodal effects on the molecular phenotypes investigated. Most e/sQTL resided within 100 kb of the affected genes or splicing junctions. We pinpoint candidate causal STRs and SVs associated with the expression of *SLC13A4* and *TTC7B* and alternative splicing of a lncRNA and *CAPP1*. We provide a catalog of STRs and SVs for taurine cattle and show that these variants contribute substantially to gene expression and splicing variation.

## Teaser

Structural variants (SVs) and short tandem repeats (STRs) are significant sources of genetic variation; however, genotyping them is challenging. Bhati *et al*. report a catalogue of STRs and SVs for taurine cattle and explore their impacts on gene expression and splicing variation in testis tissue, finding that these variant types warrant scrutiny in association testing efforts as they are in incomplete linkage disequilibrium with adjacent SNPs but show twofold enrichment in both expression and splicing quantitative trait loci.

## Introduction

Genome-wide association studies (GWAS) and expression and splicing QTL (e/sQTL) mapping establish links between genotype and (molecular) phenotype (Visscher *et al.* 2012; Yang *et al.* 2013; Littlejohn *et al.* 2016; Lopdell *et al.* 2017; Sinnott-Armstrong *et al.* 2021). These approaches typically rely on SNP and small insertion and deletion (Indel, smaller than 50 bp) markers because they can be genotyped easily and accurately with short sequencing reads using reference-guided approaches. Complex DNA variations such as structural variants (SVs, larger than 50 bp) or short tandem repeats (STRs) are often neglected for GWAS and e/sQTL mapping because they are challenging to genotype. However, it becomes increasingly apparent that SVs and STRs contribute substantially to trait variation (Baker 2012; Sudmant *et al.* 2015; Abel *et al.* 2020; Bertolotti *et al.* 2020; Collins *et al.* 2020).

SVs can be classified into deletions (DEL), duplications (DUP), insertions, inversions (INV), translocations, segmental DUP, mobile element insertions, or complex rearrangements, which may be a combination of multiple types (Ho *et al.* 2020; Belyeu *et al.* 2021). Tandem repeats are consecutive repeats of units ranging from 1 bp to several kb (Gymrek 2017). STRs specifically refer to repeats of a motif between 1 and 6 bp in length, e.g. AGC_7_ indicates that a trinucleotide (3 bp) AGC motif is repeated 7 times, yielding a total length of 21 bp. Polymorphic STRs can vary in length due to a contraction or expansion of the repeat motif. These variants can arise due to recombination errors, insertions of mobile genetic elements, slippage during DNA replication, or imperfect DNA repair (Ellegren 2000; Sun *et al.* 2012; Escaramís *et al.* 2015).

Historically, SVs and STRs had been genotyped using microarray- and PCR-based methods. The resulting genotypes were used to validate parentage, construct genetic linkage maps, assess genetic diversity, and map QTL (Machugh *et al.* 1994; Ihara *et al.* 2004; Wang *et al.* 2007; McClure *et al.* 2012). However, these methods investigate only a small number of polymorphic SVs and STRs. Exhaustive genome-wide discovery and genotyping of SVs and STRs have become feasible through advancements in short-read sequencing and variant detection methods (Willems *et al.* 2014; Sudmant *et al.* 2015; Saini *et al.* 2018; Audano *et al.* 2019; Abel *et al.* 2020; Collins *et al.* 2020). Yet, there are only few studies that identified SVs using whole-genome sequencing data in cattle (Boussaha *et al.* 2015; Chen *et al.* 2017; Mesbah-Uddin *et al.* 2018; Kommadath *et al.* 2019; Lee *et al.* 2023). To the best of our knowledge, STRs have not been profiled systematically in different cattle breeds using whole-genome sequencing data, as there is only 1 study that characterized 60,106 STRs in 5 Holstein (HOL) cattle (Xu *et al.* 2017).

It is well known from investigations in species other than cattle that STRs and SVs contribute substantially to complex traits and diseases through mediating gene expression and splicing (Cao *et al.* 2020; Scott *et al.* 2021; Vialle *et al.* 2022). For instance, an intronic AAGGG expansion in the *RFC1* gene encoding replication factor C1 is associated with cerebellar ataxia with neuropathy and bilateral vestibular areflexia syndrome in humans (Rafehi *et al.* 2019). Analyses of the human Genotype-Tissue Expression (GTEx) data showed that SVs were the lead variants in 2.66% *cis*-eQTL (Scott *et al.* 2021) and revealed many STRs affecting gene expression (Fotsing *et al.* 2019). A recent study by Hamanaka *et al.* (2023) showed that tandemly repeated motifs of up to 20 bp contribute substantially to alternative splicing and thereby phenotype variation (Hamanaka *et al.* 2023). Recent efforts from the cattle GTEx project (Liu *et al.* 2022) have revealed noncoding SNPs and Indels that impact splicing and expression variation in multiple tissues. However, they did not explore the roles of SVs and STRs. To date, the contributions of SVs and STRs to gene expression and splicing variation are largely unknown in cattle. Therefore, we generated a catalog of polymorphic STRs and SVs from 183 whole-genome sequenced cattle and assessed the impact of these variant types on gene expression and splicing in testis transcriptomes of 75 mature bulls. Finally, we pinpoint candidate causal STRs and SVs that modulate the expression and splicing of genes in testis tissue.

## Materials and methods

### Alignment and variant calling (SNPs and Indels)

We used paired-end (2 × 150 bp) whole-genome sequencing data of 183 individual cattle (mean coverage 12×) from the Brown Swiss (BSW), Original Braunvieh (OB), Grauvieh (GV), HOL, and Fleckvieh (FV) breeds and their crosses. Reference-guided alignment and variant discovery were performed as described in Lloret-Villas *et al.* (2021). Breed information and coverage for all animals, along with their accession numbers, can be found in Supplementary Table 1 in File 2. In brief, we aligned reads that passed quality control to the ARS-UCD1.2 reference genome using the MEM algorithm of the Burrows-Wheeler Alignment (BWA) software (Li 2013) with option -M. Read duplicates were marked with the MarkDuplicates module from the Picard Tools software suite (Broad Institute 2021). Subsequent discovery and genotyping of SNPs and Indels were performed with GATK HaplotypeCaller (version 4.1) (Depristo *et al.* 2011). We filtered the variants with hard filtration settings recommended by GATK to retain high-quality SNPs and Indels. Finally, we imputed sporadically missing genotypes with Beagle (version 4.1) (Browning and Browning 2016) and retained variants with minor allele frequency > 0.05 for downstream analysis.

### Building reference STRs

A previously proposed HipSTR (Willems *et al.* 2017) workflow (https://github.com/HipSTR-Tool/HipSTR-references/tree/master/mouse) was applied to compile a set of reference STRs from the soft-masked ARS-UCD1.2 reference genome [available from Ensembl (v. 104)]. Briefly, we ran the Tandem Repeat Finder (TRF) software for each chromosome with settings 2,7,7,80,10,5,500 -h -d -l 6 -ngs (Benson 1999). We retained repeats with a motif size between 1 and 6 bp, merged overlapping repeats, and finally kept sites with high scores according to motif size as implemented in the trf_parser.py utility. STRs that are not within 10 bp from another STR were retained.

### STR genotyping

The STRs were genotyped in the cohort of 183 cattle using the default mode of the HipSTR software tool (Willems *et al.* 2017). The resulting VCF file was filtered using the filter_vcf.py script from HipSTR, with options --min-call-qual 0.8, --max-call-flank-indel 0.20, and --min-loc-depth 5x. We kept only STRs with genotyping rate higher than 60% and at least 1 bp difference. Furthermore, the minimum STR length was established at 11 base pairs, which means that mononucleotide STR required a minimum of 11 copies.

### SV calling

We applied the smoove workflow to discover and genotype SVs from short sequencing reads (Pedersen *et al.* 2020). This approach extracts split and discordant reads from each bam file using samblaster (Faust and Hall 2014). These reads are then further filtered using lumpy (Layer *et al.* 2014) based on several quality metrics. The filtered reads were subsequently used to genotype SVs in each sample separately. The sample-specific SV calls were merged to obtain a set of SVs that segregate in the cohort. Each sample was then regenotyped for the common set of SVs using SVTyper (Chiang *et al.* 2015), and Duphold (Pedersen and Quinlan 2019) was run to add depth fold change. A single joint VCF file was eventually generated that contained DEL, DUP, INV, and breakends (BND). We retained only SVs that were longer than 50 bp, for which the breakpoints were precisely known, and that were supported by at least 1 split read. We kept DUP based on average DHFFC (fold change of the variant depth relative to flanking regions) scores as het > 1.25 and homo alt > 1.3 and DEL with DHFFC het < 0.70 and DHFFC homo < 0.50. INV were kept if their quality score was above 100. If multiple SVs were reported for the same location, we kept the variant with the highest quality score.

### Annotation of variants

Both STRs and SVs were annotated according to the Ensembl annotation (v. 104) of the bovine genome in a hierarchical manner using BEDTOOLS intersect (Quinlan and Hall 2010) (exon > intron > promoter > intergenic). The promoter was defined as the region located within 5 kb upstream of the gene start (5′ end). We assessed if exonic SVs overlap the whole gene or if they overlap only partially as proposed by Collins *et al.* (2020). SNPs and Indels were annotated with the Variant Effect Predictor (VEP) tool (McLaren *et al.* 2016) based on the Ensembl annotation of the bovine genome (version 104). The most severe consequence for each variant was then assigned to exon, intron, promoter, and intergenic regions as above.

### Population structure and linkage disequilibrium

We used Plink1.9 (Purcell *et al.* 2007) to calculate the principal components of genomic relationship matrices constructed from SNPs/Indels, SVs, or STRs. We used Bcftools (Danecek *et al.* 2021) to extract all SNPs and Indels within 50 kb of SVs or STRs. For each SV and STR, we calculated linkage disequilibrium (LD) as the squared Pearson correlation coefficient (*R*^2^) with the dosage of each surrounding SNP or Indel (maf > 0.05) where dosage is 0 for the 0/0, 1 for the 0/1, and 2 for the 1/1 genotype (Fotsing *et al.* 2019).

### Preprocessing RNA-seq data and alignment

Total RNAs of testis tissue from 76 mature bulls that are a subset of the 183 bulls used to profile STRs and SVs were available from a previous study (Kadri *et al.* 2021). The stranded paired-end reads were trimmed for adapter sequences, low-quality bases, and poly-A and poly-G tails with fastp (Chen *et al.* 2018). The filtered reads were aligned to the ARS-UCD1.2 reference genome and the Ensembl gene annotation (v.104) using STAR (version 2.7.9a) with options -- twopassMode, --waspOutputMode, and --varVCFfile (Dobin *et al.* 2013).

### Gene expression quantification

Gene-level expression [in transcript per million (TPM)] was quantified with the QTLtools quan function with default settings (Delaneau *et al.* 2017). Raw read counts were obtained with FeatureCounts (Liao *et al.* 2014). We retained genes that had expression values >0.2 TPM in at least 20% of samples and >6 reads in at least 20% of samples. A PCA was conducted using log_2_(TPM + 1) transformed expression values. One sample was excluded as it appeared as an outlier in the PCA. Finally, TPM values were quantile normalized and inverse normal transformed across samples per gene using the R package RNOmni (McCaw *et al.* 2020).

### Splicing quantification

We used RegTools (Cotto *et al.* 2023) and LeafCutter (Li *et al.* 2018) to quantify intron excision ratios. First, we filtered the STAR-aligned bam files for uniquely aligned and wasp-filtered reads (tag vW:i:1) (van de Geijn *et al.* 2015). Next, exon–exon junctions were obtained using RegTools with option -a 8 -m 50 -M 500000 -s 1. Finally, introns were clustered with a modified version of the leafcutter_cluster.py script provided by the human GTEx Consortium (The GTEx Consortium 2020). The script additionally filters introns without any read counts in >50% of samples and insufficient variability. Finally, the filtered intron counts were normalized using the prepare_phenotype_table.py script from LeafCutter and converted to BED format with the start/end position corresponding to the first position of 5′ of intron cluster.

### Covariates for e/sQTL analysis

To account for hidden confounders that might cause variance of gene expression or splicing, we applied the probabilistic estimation of expression residuals (PEER) (Stegle *et al.* 2012). The top 3 principal components of a genomic relationship matrix that was calculated based on LD-pruned (--indep-pairwise 50 10 0.1) SNPs using Plink1.9 (Purcell *et al.* 2007) were used to account for population structure. The influence of covariates on gene expression and splicing was quantified with the variancePartition R package (Hoffman and Schadt 2016).

### e/sQTL mapping

We used the difference in length between reference and alternate (computed from the sum of the GB format tag in the output VCF file from HipSTR) alleles as dosage for the STRs for eQTL mapping (Fotsing *et al.* 2019; Jakubosky *et al.* 2020). To minimize the effect of outlier STRs, we converted the genotypes to missing if they were not observed in at least 2 samples. We kept sites with >80% genotyping rate. To prevent the removal of multiallelic sites by QTLtools, we replaced the alternate allele field of the VCF file with the string “STR.” Furthermore, the GT field (genotype) was substituted with dosage values. Genotypes of SVs, SNPs and Indels, were also converted to dosages (0/0 to 0, 0/1 to 1, and 1/1 to 2). Genotypes at each variant position were normalized so that the effect size can be compared across the different variant types. All these changes were implemented using custom Python scripts. We performed *cis*-eQTL mapping between expressed genes and all variants in *cis* (±1 Mb) with QTLtools using the *cis* permutation mode (1,000 permutations) and accounting for covariates (5 PEER factors, 3 principal components (PC), RNA integrity number (RIN), and age). To account for multiple testing per molecular phenotype (Genes), we used the Storey and Tibshirani false discovery rate procedure that was implemented with the R/qvalue package on beta-approximated *P*-values (eGene) as described by Delaneau *et al.* (2017). This approach resulted in genes (eGenes) that had at least 1 significant eVariant and threshold *P*-values for all genes. Finally, we performed conditional analyses using QTLtools with threshold *P*-values to identify all significant independent eVariants per eGene that were used for all subsequent comparison.

*cis*-sQTL mapping was performed as described above with QTLtools using the *cis* permutation mode and accounting for covariates (5 PEER factors, 3 PC, RIN, and age). We employed grouped permutations (--grp_best option) to collectively calculate an empirical *P*-value across all introns within an intron cluster. Normalized intron excision ratios (the ratio of the reads defining an excised intron to the total number of reads of an intron cluster) were used as molecular phenotypes. We considered sQTL to be an sVariant per sIntron cluster pair. Significant intron clusters were annotated (candidate intron boundaries per cluster) based on the ARS-UCD1.2 gene annotation and strand match (Ensembl release 104). Intron cluster coordinates that mapped to multiple genes were considered as unannotated although the number of such intron clusters was less than 100 in each sQTL analysis.

### Properties of e/sVariants

From each sQTL/eQTL analysis, we annotated each e/sVariant type with their respective annotation category as described above. For all enrichment analyses, we used Fisher's exact test (2 sided). All plots were created in R (v 3.6.3) with ggplot2. Gene structure was plotted with the ggtranscript R package (Gustavsson *et al.* 2022), and plots were combined with patchwork (Pedersen 2023).

## Results

We used paired-end whole-genome sequencing data of 183 cattle from 5 breeds to genotype SVs, STRs, SNPs, and Indels. The average sequencing coverage was 12.8-fold, and it ranged from 5.0- to 30.4-fold.

### Reference-guided discovery of STRs

We identified 1,202,536 STRs with a motif size between 1 and 6 bp in the current *Bos taurus taurus* reference sequence (ARS-UCD1.2) spanning 24.9 Mb autosomal sequence (1.0%) (Supplementary Fig. 1 in File 1 and Table 2 in File 2). The number of STRs on each chromosome was correlated (*r* = 0.99) with chromosome length (Supplementary Fig. 2 in File 1). Mono- and hexanucleotide loci were the most and least frequent types of STRs, respectively, amounting to 35.9 and 9.8% of all identified STRs (Supplementary Fig. 1 in File 1). Repeats of A, T, and AT were most prevalent among mono- and dinucleotide STRs. GC-rich repeats (e.g. AGC) were most frequent among trinucleotide STRs (Supplementary Fig. 3 in File 1). The overall length of the STRs varied from 11 to 10,427 bp with a median size of 18 bp. The vast majority of the STRs (*n* = 1,199,357, 99.7%) were shorter than 100 bp, facilitating short-read–based genotyping.

### Genotyping of STRs

**Fig. 1.**
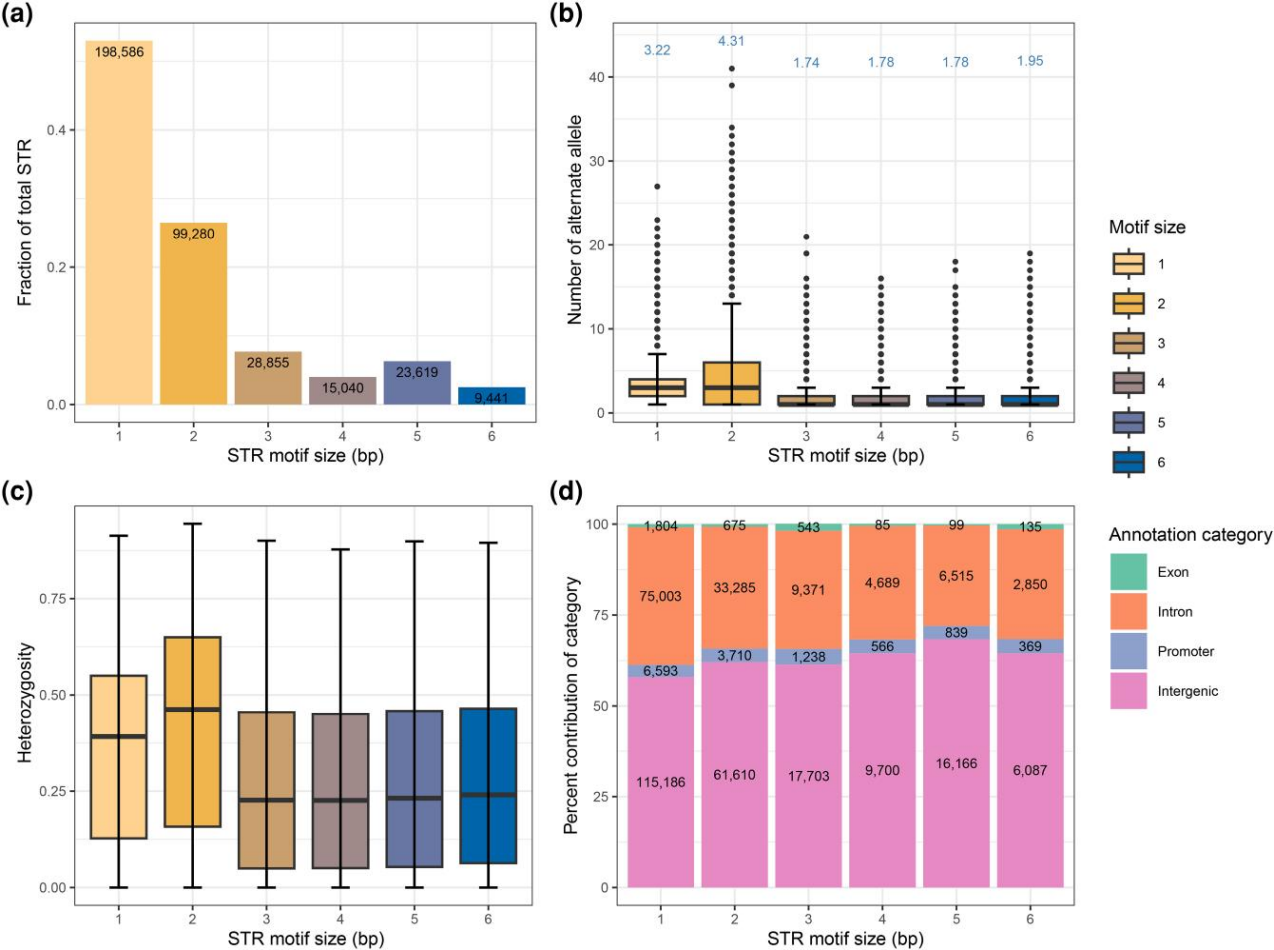
Properties of 374,822 polymorphic STRs in 183 taurine *B. taurus taurus* cattle genomes. a) Proportion and count of STRs for each motif size. b) Number of alternative alleles observed for each STR motif size. Numbers above the boxplots indicate the average number of alleles observed in the 183 cattle genomes for each motif size. c) Heterozygosity in each STR motif. d) Proportion of loci overlapping 4 annotation categories for each STR motif size. Numbers inside the stacked bars represent the total count of STRs for each annotation category.

We obtained genotypes for 794,300 autosomal STRs in 183 cattle using HipSTR (Willems *et al.* 2017), of which we retained 374,822 polymorphic loci after stringent filtering for downstream analyses (Supplementary Table 3 in File 2). We identified between 73,791 and 189,658 (average: 150,104) STRs in each cattle genome, and the number of STRs detected correlated (*r* = 0.94) with sequencing depth (Supplementary Fig. 4 in File 1). As expected, given their prevalence in the bovine reference genome, mono- (52.9%) and hexanucleotide STRs (2.5%) were respectively the most and least frequent types of polymorphic STRs (Fig. 1a). Pentanucleotide STRs were more frequent than tetranucleotide STRs. Approximately three-quarters of polymorphic STRs (*n* = 266,509, 71.1%) were multiallelic and had between 1 and 41 alternate alleles, but more than 20 alternate alleles were rarely seen (Supplementary Fig. 5 in File 1). Dinucleotide STRs had the highest number of alternative alleles among all STRs (Fig. 1b). Repeats of A and T were the most frequent classes among the mononucleotide STRs, whereas AGC and CTG repeats prevailed among trinucleotide STRs (Supplementary Fig. 6 in File 1). Heterozygosity and allelic diversity were higher for dinucleotide loci than any other type of STRs (Fig. 1b and c), possibly suggesting higher mutation rate and less purifying selection in this class.

We investigated the overlap between the STRs detected in our study and 16 STRs that had been frequently used for parentage testing [including those that had been approved by the International Society for Animal Genetics (ISAG)] (Van De Goor *et al.* 2009) (Supplementary Table 4 in File 2). Forward and reverse primer sequences of the 16 STRs were subjected to a BLASTn search against the ARS-UCD1.2 reference sequence to identify their positions, motifs, and flanking sequences. Twelve STRs from our STR reference panel had matching coordinates and motif sequences. Among them, 10 STRs were genotyped in 183 samples with allele counts ranging from a minimum of 5 to a maximum of 11, whereas the remaining 2 STRs were excluded during quality filtering.

Functional annotation showed an enrichment of STRs in intergenic regions (60.4%, *P* = 0.002, odds ratio (OR) = 1.26). STRs were depleted in exonic regions (0.89%, *P* = 0.003, OR = 0.36) and promoter regions (3.55%, *P* = 0.027, OR = 0.66) (Fig. 1d; Supplementary Fig. 7 in File 1). The proportion of STRs that overlapped exons was highest for tri- (1.9%) and hexanucleotide (1.4%) motifs (Fig. 1d), which were the least heterozygous among all annotation categories (Supplementary Fig. 8 in File 1).

### SV discovery and genotyping

**Fig. 2.**
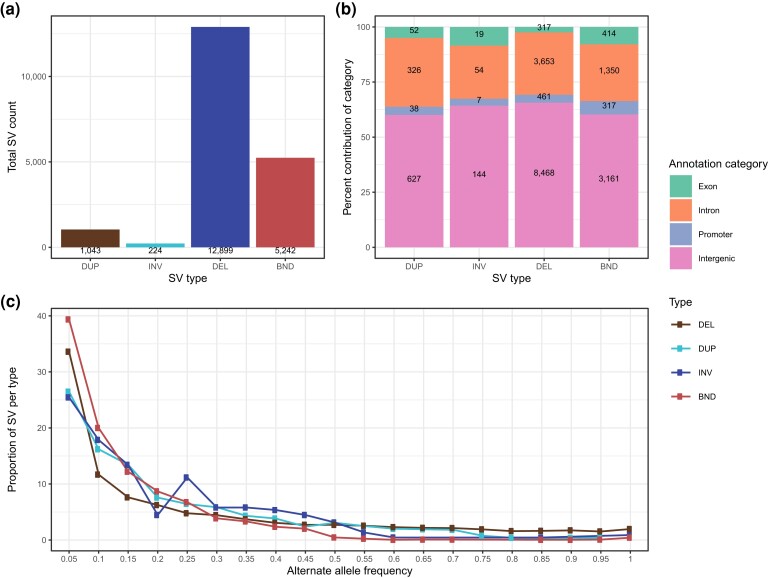
Properties of 19,420 polymorphic SVs in 183 taurine cattle genomes. a) Count of polymorphic loci for each SV type. b) Proportion of loci overlapping 4 annotation categories. The numbers inside the stacked bars represent the count of SVs for each annotation category. c) Alternative allele frequency distribution for each SV type.

We applied the smoove workflow to discover and genotype 61,806 SVs in the 183 cattle genomes, of which we retained 19,408 polymorphic autosomal loci (12,899 DEL, 1,043 DUP, 224 INV, and 5,242 SVs with unspecified BND) after stringent filtering for downstream analyses (Supplementary Fig. 2a in File 1 and Table 5 in File 2). The number of polymorphic SVs identified per chromosome was correlated (*r* = 0.94) with chromosome length (Supplementary Fig. 9 in File 1). We found between 4,259 and 6,835 SVs in each cattle genome (mean: 5,915), and this number was correlated with sequencing coverage (*r* = 0.60) (Supplementary Fig. 10 in File 1). A total of 6,728 (34.6%) SVs had minor allele frequency below 0.05 (Fig. 2c). Inspecting the length of the different SV types suggested that most (*n* = 891, 85.4%) DUP were smaller than 1 kb, whereas 3,465 (23%) DEL were larger than 1 kb (Supplementary Fig. 11 in File 1).

We annotated the SVs according to their location to assess putative functional consequences. This approach revealed that 12,400 (63.8%), 5,383 (27.7%), 823 (4.2%), and 802 (4.1%) SVs overlapped intergenic, intronic, promoter, and exonic regions, respectively (Fig. 2b). SVs partially or fully overlapped 3,863 genes. Among the SVs that overlapped exons, we identified 52 DUP (25 copy gain DUP or whole-gene DUP, 4 full exonic DUP, and 23 partial exonic DUP), 19 INV (15 whole-gene INV and 4 INV with 1 breakpoint in exon), and 317 putative loss of function DEL (162 whole-gene DEL and 155 DEL affected at least 1 exon with 1 breakpoint). We also detected 414 BND in exons. Whole-gene INV (median size 867.9 kb) were the type of exonic SV that was largest in size and lowest in number. The whole-gene INV detected encompassed 182 coding genes and 32 noncoding genes. Approximately one-third of the SVs (*n* = 7,083, 36.4%) were only present in the heterozygous state, and most of these (*n* = 4,989, 70.4%) had minor allele frequency less than 0.05 (Fig. 2c). Among these, 4,345, 2,017, 391, and 330 overlapped with intergenic, intronic, promoter, and exonic regions, respectively.

### LD and population structure of the cattle cohort

We also discovered and genotyped SNPs and Indels in the 183 animals using the GATK haplotype caller. We considered 12,222,397 SNPs and 1,317,363 Indels with minor allele frequency greater than 5% for the downstream analyses, of which 55,010 SNPs and 89,673 Indels overlapped with STRs, and 387,593 SNPs and 47,129 Indels overlapped with SVs. The large overlap between SNP, SV, and STR variants is possibly due to nested variation but can also indicate that short sequencing reads are unable to resolve complex DNA variation.

We calculated the principal components from genomic relationship matrices built with SNP, STR, and SV genotypes of the 183 cattle. All three analyses correctly separated the individuals by breed (Supplementary Fig. 12 in File 1). Due to variation in sample size, coverage, and insert size between breeds, we did not investigate within- and across-breed diversity in SVs and STRs. Next, we investigated if SVs and STRs can be tagged by SNPs/Indels. We calculated the LD between SNPs/Indels within 100 kb of each SV and STR. We observed that 40.1% of STRs (*n* = 150,393) were in high LD (*R*^2^ > 0.8) with at least 1 SNP or Indel while this fraction ranged from 3.1 to 52.2% for the different SV types (Supplementary Fig. 13a in File 1 and Table 6 in File 2). BND and DUP were poorly tagged, possibly indicating low genotyping accuracy for these loci. The LD between SNPs/Indels and STRs was consistent across the different STR types (Supplementary Fig. 13b and Table 6 in File 2).

### Properties of STRs and SVs associated with gene expression

The impact of polymorphic SVs, STRs, SNPs, and Indels on gene expression was investigated in a subset of 75 sequenced bulls that also had testis RNA-sequencing data. We performed *cis*-eQTL mapping between 19,415 expressed genes and 12,093 SVs, 271,450 STRs, and 13,494,075 SNPs and Indels that had minor allele frequency greater than 5% in the 75 bulls. Five eQTL analyses were conducted, i.e. for SNPs and Indels, SVs, STRs, and jointly for SVs and STRs (SV–STR), and all (ALL) variants to assess the contribution of different types of DNA variation to gene expression.

An eQTL mapping with 13,494,075 ALL variants revealed 6,627 eGenes associated with 7,398 unique eVariants (25 SVs, 514 STRs, 964 Indels, and 5,902 SNPs). Both SVs (OR = 3.98 and *P* = 1.4 × 10^−8^) and STRs (OR = 3.6, *P* = 6.7 × 10^−125^) were enriched among the eVariants indicating that these variant types contribute disproportionally to gene expression variation. The SV–STR eQTL mapping revealed 5,641 eGenes associated with 5,971 unique eVariants (Table 1). The subsequent separate variant type eQTL mapping revealed 6,550, 1,798, and 5,669 eGenes with 7,303, 1,391, and 5,995 unique eVariants, respectively, when only SNPs/Indels, SVs, and STRs were considered (Table 1). A total of 1,514 eGenes overlapped between the 5 eQTL analyses (Supplementary Fig. 14 in File 1). Most eGenes (3,379) were shared between the separate eQTL analyses, but 1,420 eGenes were shared only between SNPs and Indels and ALL suggesting that many eGenes are only associated with SNPs and Indels. A larger proportion of eSV (24.1% of eSV) and eSTR (8.0% of eSTR) than eSNV/eIndel (2.9% of eSNP/Indel) were associated with the expression of multiple eGenes (Table 1).

**Table 1.** Overview of *cis*-eQTL detected in 75 testis transcriptomes.

Type	Total variants	eGenes	eVariant (% total variants)	eVariant (affecting > 1 eGene)	eQTL (eVariant–eGene pair)
SNP and Indel	13,210,530	6,550	7,303 (0.05%)	153	7,665
SV	12,093	1,798	1,391 (11.5%)	336	1909
STR	271,450	5,669	5,995 (2.2%)	485	6,572
SV–STR	283,545	5,641	5,971 (2.1%)	465	6,525
ALL	13,494,075	6,627	7,398 (0.05%)	146	7,552

**Fig. 3.**
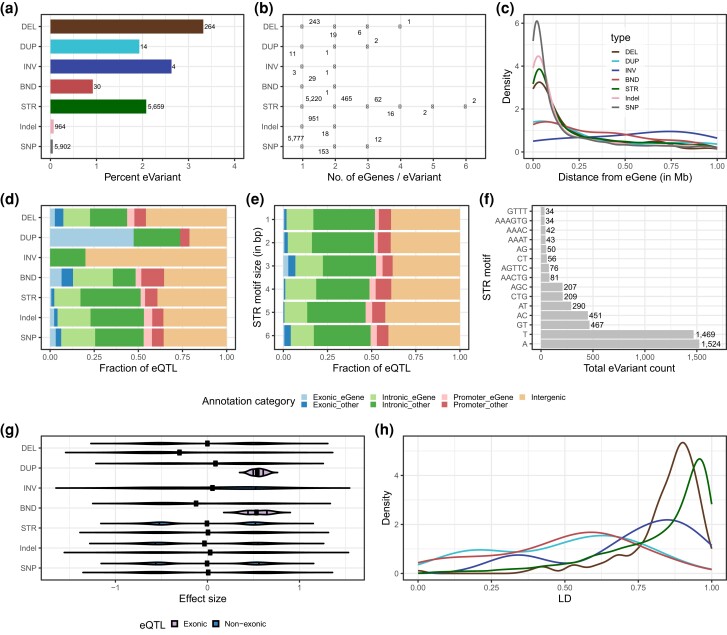
Properties of eSVs and eSTRs from the SV–STR eQTL mapping. eSNPs and eIndels are added from the ALL eQTL mapping. a) Percentage of unique eVariants for each variant type. The count of eVariants per type is shown next to each bar. b) Number of eGenes affected by each type of eVariants. c) Distribution of the absolute distance between eVariants and eGenes (5′-UTR or TSS). d) Proportion of eQTLs from different annotation categories in each variant type, such as those overlapping intergenic regions or overlapping exons, promoters, or introns of their corresponding eGenes or other genes. e) Proportion of eQTLs from different annotation categories in each STR type such as those overlapping intergenic regions or overlapping exons, promoters, or introns of their corresponding eGenes or other genes. f) Total count of the most frequent STR motifs (>30 observations) among eSTR. g) Distribution of effect size of eQTL per type based on exonic [overlap with an exon (exonic) of their eGene or other genes] or nonexonic category. h) Distribution of maximum LD (*R*^2^) per variant for each eVariant type.

The contribution of STRs and SVs to gene expression variation was quantified based on results from the SV–STR eQTL analysis (Supplementary Table 7 in File 2). The eVariants were more strongly enriched for SVs than STRs (312 eSV, OR = 1.2, *P* = 3.3 × 10^−4^), but most eVariants were STRs (5,659 eSTRs out of 5,971 eVariants). Among the different SV types, DEL were enriched (264 eDEL, OR = 1.6, *P* = 2.9 × 10^−12^), and BND were depleted (30 eBND, OR = 0.4, *P* = 3.3 × 10^–7^) among the eVariants compared to STRs (Fig. 3a; Supplementary Table 8 in File 2). The proportion of eVariants associated with multiple eGenes was higher for eDUP (21.4%) than eSTRs (9.6%). Overall, eDUP affected on average 1.35 eGenes (eSV 1.12 eGenes) whereas eSTR and eSNP and eIndel affected 1.11 and 1.01 eGenes, respectively. The maximum number of eGenes per eVariant was larger for STR (*n* = 6) than any other variant type (Fig. 3b).

We examined the distance between eVariants and eGenes [5′-UTR or transcription start site (TSS)] and found that most eSVs and eSTRs were located within 250 kb of eGenes (Fig. 3c) but eBND (48.3%) and eINV (80%) were more distant (>250 kb) from their eGenes (Supplementary Table 9 in File 2). Overall, 19.9% eVariants (*n* = 1,194) overlapped with their eGenes; 64 (0.9%), 166 (2.5%), and 964 (14.7%) overlapped with exons, promoters, and introns, respectively. Most eQTLs were in introns (48.1%) or intergenic regions (39.4%). eDUP were enriched in exons of their eGenes (OR = 105.1, *P* = 4.0 × 10^−4^) while eDEL were enriched in the exons of other genes (OR = 2.89, *P* = 9.2 × 10^−4^). In contrast, eSTRs were depleted in the exons of their eGenes (OR = 0.1, *P* = 3.4 × 10^−4^) and other genes (OR = 0.4, *P* = 6.2 × 10^−4^) (Fig. 3d; Supplementary Table 10 in File 2). These results suggest that eDUP impact gene expression by increasing the copy of their eGenes, which agrees with previous research (Shaul 2017). The highest proportion of eSTRs overlapping with exons of their eGenes (17 eSTRs) or other genes (24 eSTRs) had a trinucleotide repeat motif (Fig. 3f). Such STRs are likely to be more tolerated and less selected against than those compromising the triplet codon structure. Most trinucleotide eSTRs in exons had GC-rich repeat motifs (CGG, CTG, CCG, and AGC).

Exonic eDUP predominantly increased gene expression, while exonic eDEL mostly decreased gene expression. All other eVariant types exhibited a bimodal effect size distribution. We then explored the LD between eSTRs and eSVs and nearby SNP/Indel. More than three-quarters (78.4%) of the eDEL and two-thirds of the eSTR (65.9%) were in high LD (*R*^2^ > 0.8) with surrounding SNP/Indel. In contrast, eDUP and eBND were poorly tagged by SNP/Indel.

We found that 92.2% of the eGenes were protein-coding genes, 4.5% were long noncoding RNA (lncRNA), 0.98% were pseudogenes, and 1.8% were other genes (i.e. genes that are not classified in the above 3 categories) (Supplementary Fig. 15 in File 1). We observed a similar distribution of eGenes across all eVariant types except for eINV.

**Fig. 4.**
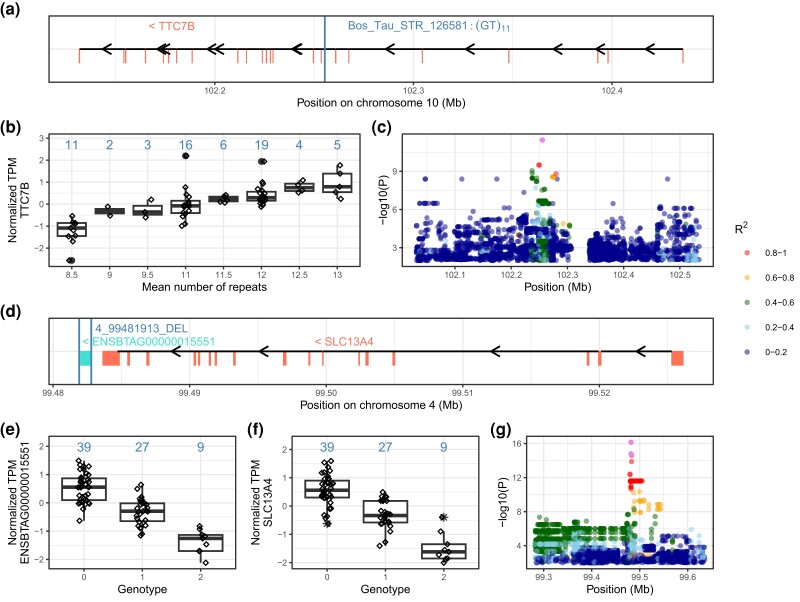
Candidate eSTR a–c) and eSV d–g) associated with eGene expression. The eSTR Bos_Tau_STR_126581 is a GT dinucleotide that repeats 11 times in the reference sequence and between 8.5 and 13 times in the 75 genotyped bulls. eQTL mapping revealed association between Bos_Tau_STR_126581 and *TTC7B* mRNA abundance. a) Schematic overview of the exon/intron structure of bovine *TTC7B* gene and Bos_Tau_STR_126581. The vertical line indicates the position of Bos_Tau_STR_126581, and the vertical half lines indicate exons of *TTC7B*. b) Normalized gene expression of *TTC7B* in 75 genotyped bulls in each mean dosage of eSTR. The numbers above each boxplot are the total number of animals per genotype. The mean repeat count per genotype is determined by adding the total repeat number of both alleles belonging to a genotype and then dividing this sum by 2. c) Manhattan plot of −log_10_(*P*)-values for all variants surrounding Bos_Tau_STR_126581 (pink colour) from the nominal ALL eQTL analysis. Different colors indicate the pairwise LD (*R*^2^) between Bos_Tau_STR_126581 and all other variants. d) Schematic overview of *ENSBTAG00000015551* (turquoise color) and *SLC13A41* (salmon color) that are associated with a 885-bp DEL on chromosome 4 (eDEL 4_99481913_DEL). The boxes represent exons. The vertical lines indicate the position of 4_99481913_DEL. e, f) Normalized mRNA expression of *ENSBTAG00000015551* and *SLC13A41* in 75 genotyped bulls for each genotype of eDEL. The numbers above each boxplot are the total number of animals per genotype. g) Manhattan plot of −log_10_(*P*)-values for all variants surrounding 4_99481913_DEL from the nominal ALL eQTL analysis as pink colour (2 points as same eDEL corresponds to 2 genes). Different colors indicate the pairwise LD (*R*^2^) between 4_99481913_DEL and all other variants.

We identified a candidate causal eSTR (GT_11_, Bos_Tau_STR_126581, Chr10:102,255,360–102,255,381 bp) in the seventh intron of *TTC7B* encoding tetratricopeptide repeat domain 7B (Fig. 4a). The abundance of *TTC7B* mRNA (mean TPM 4.9 ± 1.3) increased with an expansion of the GT repeat motif (*P* = 3.4 × 10^−12^). This STR was the top eVariant in both the ALL and SV–STR eQTL analyses (Fig. 4b and c). A candidate causal eSV is a 885-bp DEL (Chr4:99,481,913–99,482,798 bp) encompassing *ENSBTAG00000015551* and the distal end of *SLC13A41* encoding solute carrier family 13-member 4 (Fig. 4d). The DEL reduced mRNA expression of *ENSBTAG00000015551* (mean TPM 7.2 ± 3.4, *P* = 7.1 × 10^−17^) and *SLC13A4* (mean TPM 0.9 ± 0.4, *P* = 1.6 × 10^−15^) (Fig. 4e and f). This DEL was the top eVariant in the ALL and SV–STR eQTL analyses for both genes.

### *cis*-sQTL mapping

We calculated intron excision ratios of 241,427 introns assigned to 76,083 intron clusters. More than half (*n* = 135,342, 56.0%) of the introns overlapped with 14,583 genes, but the annotation-free splicing event identification by the LeafCutter software also detected many introns that did not overlap with annotated features. The intron excision ratios were normalized for each intron and subsequently used as input phenotypes for *cis*-sQTL mapping. We mapped *cis*-sQTL with an approach that was similar to the eQTL mapping, i.e. we separately considered SNPs and Indels, SVs, STRs, SV–STR, and ALL.

The ALL sQTL mapping revealed association between 12,835 unique lead variants (sVariant) and 11,588 (15.2%) intron clusters (sIntron cluster). The 12,835 sVariants included 25 SVs, 712 STRs, 1,593 Indels, and 10,505 SNPs, and 286 of the sVariants were associated with more than 1 intron cluster. More than half of the sIntron clusters (*n* = 6,798, 58.6%) overlapped with 4,890 sGenes whereas the remaining did not overlap with annotated features. Both SVs (OR = 2.3, *P* = 2.3 × 10^−4^) and STRs (OR = 2.9, *P* = 6.4 × 10^−123^) were enriched among the sVariants when compared to SNPs and Indels. The SV–STR analysis revealed 9,065 sIntron clusters associated with 8,857 unique sVariants (Table 2). Variant type–specific sQTL analyses revealed 8,749, 1,707, and 12,683 sVariants, respectively, when only STR, SV, and SNP and Indel were considered (Table 2).

**Table 2.** Overview of *cis*-sQTL detected in 75 testis transcriptomes.

Type of variants in sQTL analysis	Total variants	sIntron clusters	Not annotated sIntron cluster	sGenes	sVariants	sQTL (sVariant–sIntron cluster pair)
SNP and Indel	13,210,530	11,452	4,748	4,831	12,683	13,003
SV	12,093	2,463	1,051	1,182	1,707	2,552
STR	271,450	8,999	3,708	3,990	8,749	10,008
SV–STR	283,545	9,065	3,755	4,001	8,857	10,083
ALL	13,494,075	11,588	4,790	4,890	12,835	13,136

We then assessed the overlap of sGenes/sIntron clusters between all sQTL analyses. Approximately half of the sIntron clusters (*n* = 6,034, 47.9%) and sGenes (*n* = 2,590, 49.1%) overlapped between the SNPs and Indels, STR, SV–STR, and ALL sQTL analyses suggesting that distinct variant types in LD tag the same splicing event (Supplementary Figs. 16 and 17 in File 1). A total of 3,126 (24.8%) sIntron clusters and 1,126 (21.3%) sGenes were shared only between SNPs and Indels and ALL suggesting that a substantial fraction of sGenes is only associated with SNPs and Indels.

### Variant properties of sSTR and sSV

**Fig. 5.**
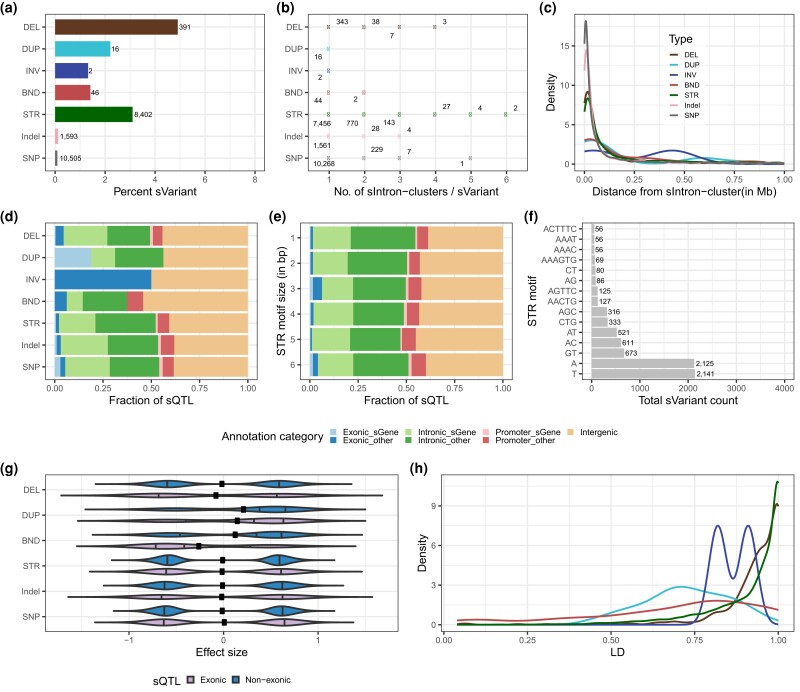
Properties of sVariants (SVs and STRs) from the SV–STR sQTL analysis. SNPs and Indels are from ALL analyses in all panels. a) Percentage of unique sVariants for each variant type. The number of sVariants per category is shown next to the bars. b) Total number of sIntron clusters per sVariant for each variant type. c) Distance between sQTL and the start position of the associated sIntron cluster for each variant type. d) Fraction of sQTL from different annotation categories in each variant type such as those overlapping intergenic regions or overlapping exons, promoters, or introns of their corresponding sGenes or other genes. e) Fraction of sQTL from different annotation categories in each STR motif size such as those overlapping intergenic regions or overlapping exons, promoters, or introns of their corresponding eGenes or other genes. f) Prevalence of the most frequent (>50) STR motif among sSTR. g) Distribution of sQTL effects. Colors differentiate between exonic and nonexonic sQTL. The dots represent the overall mean. h) Distribution of maximum LD (*R*^2^) between sQTL and SNP/Indel for different variant types.

The impact of STRs and SVs on alternative splicing was assessed based on the results from the SV–STR sQTL analysis (Supplementary Table 11 in File 2). We observed that DEL were more likely to be sVariants than STRs (4.9% of DEL, OR = 1.6, *P* = 1.7 × 10^−17^) (Fig. 5a; Supplementary Table 12 in File 2). Conversely, BND (OR = 0.4, *P* = 1.3 × 10^−9^) were less likely to be sVariants compared to STRs. We further examined how many intron clusters are affected by an sVariant. A similar proportion of sDEL (12.2%) and sSTRs (11.2%) were associated with multiple sIntron clusters whereas this fraction was considerably lower or negligible for all other types of sVariants (Fig. 5b). Between 62 and 81% of the sVariants were located within 100 kb of their sIntron cluster (Fig. 5c; Supplementary Table 13 in File 2).

Most of the sQTL overlapped with either introns (49.7%) or intergenic regions (41.0%) but only few with promoter (6.9%) and exons (2.3%). Interestingly, sDEL were enriched in exons and depleted in introns of other genes, whereas sDUP showed enrichment in exons of sGenes (Supplementary Table 14 in File 2). On the other hand, sSTRs were depleted in exons of other genes, but they were enriched in introns of other genes (Supplementary Table 14 in File 2). We observed a high proportion of trinucleotide sSTRs among those that overlapped exons. These trinucleotide sSTRs were GC rich (Fig. 5f). Most sQTL showed bimodal effects. A bimodal effect size distribution in splicing variation encompassing both positive and negative effects is associated with variation in the relative abundance of transcripts between different genotypes (Garrido-Martín *et al.* 2021). sDUP had slightly positive effects on splicing phenotypes, which may indicate a relatively higher abundance of the transcript associated with the DUP (Fig. 5g). The vast majority of sDEL (94.0%) and sSTRs (84.3%) were in high LD (*R*^2^ > 0.8) with surrounding (±50 kb) SNP/Indel, but sDUP (31.5%) and sBND (39.5%) were less frequently tagged (Fig. 5h).

Finally, we compared genes and molecular QTL (eQTL and sQTL as gene–variant pair) from both the eQTL and sQTL SV–STR analyses (Supplementary Fig. 18 in File 1). This comparison revealed that 1,988 genes and 505 QTL overlapped between both analyses. Out of the 505 shared QTLs, 479 were due to STR, while 23 were due to DEL (Supplementary Fig. 19 in File 1). The eQTL that were also sQTL mainly regulated expression due to alterations in gene transcript level abundance, and these changes were mainly modulated by STR and DEL.

**Fig. 6.**
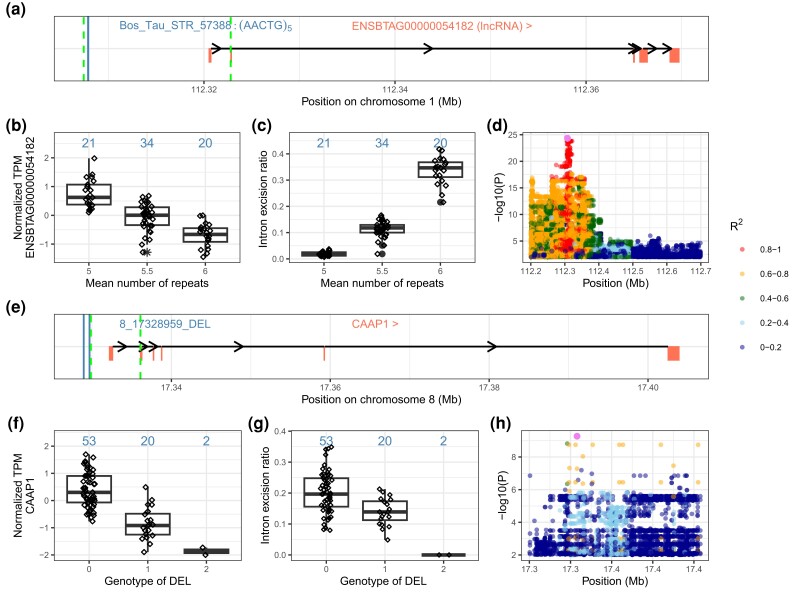
Two candidate causal sSTR a–d) and sSV f–h). The sSTR Bos_Tau_STR_57388 on chromosome 1 is associated with *ENSBTAG00000054182* splicing. a) Schematic overview of *ENSBTAG00000054182.* Bos_Tau_STR_57388 (vertical straight line) is upstream of lncRNA *ENSBTAG00000054182*, and it is associated with the splicing junction spanning from Chr1:112,307,410 to 112,322,799. Intron/splice junction boundaries are indicated with dotted lines. b) Normalized *ENSBTAG00000054182* expression and c) intron excision ratio for different sSTR genotypes. The numbers above each boxplot are the total number of animals per genotype. The mean repeat count per genotype is determined by adding the total repeat number of both alleles belonging to a genotype and then dividing this sum by 2. d) Manhattan plot of nominal ALL sQTL result surrounding the sSTR (pink colour). Different colors indicate the pairwise LD (*R*^2^) between Bos_Tau_STR_57388 and all other variants. A candidate sSV (8_17328959_DEL) on chromosome 8 is associated with alternative *CAAP1* splicing. e) Schematic overview of *CAAP1* gene. A promoter DEL “8_17328959_DEL” (vertical straight line) is associated with excision ratios of splicing junction Chr8:17,329,855–17,336,097. Intron boundaries are indicated with dotted lines. f) Normalized *CAAP1* expression and g) intron excision ratio for the different sSV genotypes.The numbers above each boxplot are the total number of animals per genotype. h) Manhattan plot of nominal ALL sQTL result surrounding sDEL (pink colour). Different colors indicate the pairwise LD (*R*^2^) between sDEL and all other variants.

Among the sQTL, we identified a candidate causal sSTR (AACTG_5_, Bos_Tau_STR_57388, Chr1:112,307,866–112,307,890 bp) upstream of the lncRNA *ENSBTAG00000054182* (Fig. 6a). An expansion of Bos_Tau_STR_57388 (the inserted motif AAATG differed slightly from the reference motif AACTG) was associated with a splicing junction (Chr1:112,307,410–112,322,799, *P* = 4.5 × 10^−25^) in both SV–STR and ALL sQTL analyses. This splicing junction extends from upstream the lncRNA to the first intron of the lncRNA (Fig. 6a), and its intron excision ratio increased with an expansion of the repeat motif (Fig. 6c and d). The expression of *ENSBTAG00000054182* (mean TPM 5.1 ± 1.5) decreased with the insertion of an additional repeat unit (Fig. 6b). Bos_Tau_STR_57388 was also the top eVariant for *ENSBTAG00000054182* in the SV–STR eQTL analysis (*P* = 2.3 × 10^−15^) but not in ALL eQTL analysis where a SNP (Chr1:112,315,134 bp) in LD (*R*^2^ = 0.93) was the top eVariant.

A candidate sSV is a 729-bp DEL on chromosome 8 (Chr8:17,328,959–17,329,688), which resides in the promoter region of *CAAP1* encoding caspase activity and apoptosis inhibitor 1 (Fig. 6e). The DEL was associated (*P* = 2.8 × 10^−11^) with reduced excision ratios of a splicing junction (Chr8:17,329,855–17,336,097) overlapping *CAAP1* (Fig. 6f and h) and expression of *CAAP1* (mean TPM 26.4 ± 2.8) (Fig. 6b). This sDEL was also the top eVariant for *CAAP1* in the ALL eQTL and SV–STR eQTL (*P* = 5.1 × 10^−12^).

## Discussion

We generated a catalog of bovine polymorphic STRs, which contain motifs that vary in size, but some may also contain variation between the repeat motifs. A large number of cattle from different breeds enabled us to genotype 6-fold more STRs compared to a previous study (374,821 vs 60,661) that considered only 5 HOL cattle genomes (Xu *et al.* 2017). Three-quarters of the STRs genotyped in our study were multiallelic, which agrees with previous studies in cattle, pigs, and humans (Willems *et al.* 2014; Xu *et al.* 2017; Wu *et al.* 2021). We also genotyped almost 20k SVs. The majority of both SVs and STRs were in introns and intergenic regions likely because coding regions are less tolerant to variants affecting several bases. We also detected SVs and STRs that overlapped exonic regions, but half of the exonic SVs were only present in the heterozygous state, which may indicate that some of them manifest deleterious phenotypes in the homozygous state. However, even deleterious SVs can persist and increase in frequency over time due to drift or pleiotropic effects and balancing selection, such as a 660-kb DEL in Nordic red cattle (Kadri *et al.* 2014). Deleterious SVs in less conserved genes may be evolutionarily less constrained (Mesbah-Uddin *et al.* 2018). We also observed a high proportion of tri- and hexanucleotide STR in exonic regions possibly suggesting that nontriplet STRs are less tolerated and might be under negative selection (Willems *et al.* 2014).

We observed more than twice the number of DEL compared to other SV types likely because they are easier to identify from short-read sequencing data (Mahmoud *et al.* 2019). Only half of the STRs and DELs are in high LD with SNPs and Indels (*R*^2^ > 0.8). The LD between SNPs and other types of SVs such as BND, DUP, and INV is even lower, which could be due to incorrect genotyping, alignment error, or their occurrence in complex regions such as segmental DUP. Thus, the direct genotyping of these variants is required to enable powerful association studies.

In the cattle breeding industry, genomic prediction is routinely applied to estimate breeding values. This estimation typically relies on dense genotypes obtained from SNP arrays or imputed sequence variants but does not consider an exhaustive set of STRs and SVs. Lee *et al.* (2023) added 372 SVs to the custom content of a frequently applied microarray to enable genotyping at the population scale. Our study confirms that such efforts are warranted, as SVs and STRs are only partially tagged by adjacent SNPs. Particularly intriguing are also SVs and STRs that are associated with gene expression and splicing variation, as they potentially impact phenotypes. Considering such SVs and STRs and those exhibiting limited or no LD with genotyped SNPs carries the potential to improve the accuracy of genomic predictions and offers insights into the molecular underpinnings of QTL (Lee *et al.* 2021; Leonard *et al.* 2023).

Our results confirm that sequencing coverage and insert size have profound impacts on the genotyping of SVs and STRs (Willems *et al.* 2017; Lee *et al.* 2023). We applied stringent filters to retain only high-confidence SVs. This approach likely removed some true large and complex SVs and STRs from our data. Long sequencing reads and pangenome integration enable to reliably detect large and complex SVs and STRs (Leonard *et al.* 2022; Talenti *et al.* 2022). However, long-read sequencing is still too costly when applied at population scale. Future studies could utilize a combination of long-read sequencing and pangenome integration with short-read sequencing data to identify and genotype the full spectrum of genetic variants at the population scale (Chaisson *et al.* 2019; Ebert *et al.* 2021).

Our study examines impacts of polymorphic SVs and STRs on gene expression and splicing variation in bovine testis tissue. Further research is needed to extend this analysis to additional tissues as gene regulation is predominantly tissue specific (Gamazon *et al.* 2018). Regardless, our eQTL and sQTL analyses showed that SVs and STRs have profound impacts on the transcriptional profile in testis. We found that each eSV affects on average 1.11 nearby genes with most of this contribution arising from DUP. However, this value is lower than the 1.82 genes in *cis* per eSV reported recently in humans, where major contributions were from multiallelic copy number variants (mCNV) and DUP (Scott *et al.* 2021). In our study, CNV are part of the DUP category. This difference likely indicates that our study had less power to detect s/eQTL because our variant catalog (61,668 SVs in human vs 19,408 SVs here) and sample size (643 individuals with 48 tissues vs 75 individuals with 1 tissue) were considerably smaller. Our results confirm that e/sDUP in exonic and nonexonic regions mostly increase gene expression whereas e/sDEL decrease gene expression (Chiang *et al.* 2017; Scott *et al.* 2021). An increased expression associated with an e/sDUP is frequently due to either DUP of the entire gene or exon or its regulatory regions. It is possible that some of the eSVs and eSTRs detected in our study are associated with semen quality and male fertility. Further investigations are needed to explore the integration of molecular QTL cohorts and GWAS cohorts through transcriptome-wide association testing. This approach holds the potential to reveal SVs and STRs associated with specific traits (Mapel *et al.* 2023).

Our analysis showed that most e/sSTRs and e/sSVs were in intronic regions rather than intergenic regions, which contrasts with their overall distribution along the genome. This pattern agrees with the position of human e/sSTRs and e/sSVs (Chiang *et al.* 2017; Fotsing *et al.* 2019; Jakubosky *et al.* 2020). Our study thus confirms the importance of noncoding SVs and STRs in regulating gene expression and splicing (Jakubosky *et al.* 2020). Intronic and intergenic regions can contain regulatory elements that modulate splicing and gene expression via change in nucleosome positioning, open chromatin structure, RNA-binding protein, or DNA methylation (Fotsing *et al.* 2019; Ho *et al.* 2020; Vialle *et al.* 2022). Nearly half of the intron clusters detected in our study could not be annotated with the current cattle annotation (Ensembl 104). The FANTOM5 consortium revealed significant overlap of TSS to STR loci, which are unassigned to any known genic or enhancer regions in humans (Grapotte *et al.* 2021). Most of these TSS overlapping with STRs are responsible for initiating noncoding RNAs in humans. Similarly, a candidate causal sSTR detected in our study was associated with the splicing of the lncRNA *ENSBTAG00000054182*, which produces a transcript that is not included in the current Ensembl annotation. This further emphasizes the need for an improved bovine annotation, particularly with respect to noncoding elements of the genome such as lncRNAs. Although the association of expression and splicing variation with STRs and SVs in e/sQTL studies does not necessarily provide the underpinning molecular mechanism of action, these variants contribute significantly to complex trait variation (Xiang *et al.* 2022).

## Data Availability

Short paired-end whole-genome sequencing reads from 183 cattle from 5 breeds and whole-genome RNA-sequencing data from 75 cattle are available at European Nucleotide Archive database (https://www.ebi.ac.uk/ena/browser/home) with the study accessions PRJEB28191, PRJEB46995, and PRJNA238491. The primary output files and raw data info including accession numbers of the raw data, reference STR, genotyped STR, and SV are made available through Zenodo (https://doi.org/10.5281/zenodo.8274665). All scripts and workflows are available online: https://github.com/Meenu-Bhati/SV-STR. Supplemental material available at figshare: https://doi.org/10.25386/genetics.24038517.

## References

[iyad161-B1] Abel HJ, Larson DE, Regier AA, Chiang C, Das I, Kanchi KL, Layer RM, Neale BM, Salerno WJ, Reeves C, et al Mapping and characterization of structural variation in 17,795 human genomes. Nature 2020;583(7814):83–89. doi:10.1038/s41586-020-2371-0. 32460305 PMC7547914

[iyad161-B2] Audano PA, Sulovari A, Graves-Lindsay TA, Cantsilieris S, Sorensen M, Welch AME, Dougherty ML, Nelson BJ, Shah A, Dutcher SK, et al Characterizing the major structural variant alleles of the human genome. Cell 2019;176(3):663–675.e19. doi:10.1016/j.cell.2018.12.019. 30661756 PMC6438697

[iyad161-B3] Baker M . Structural variation: the genome's hidden architecture. Nat Methods. 2012;9(2):133–137. doi:10.1038/nmeth.1858. 22290183

[iyad161-B4] Belyeu JR, Brand H, Wang H, Zhao X, Pedersen BS, Feusier J, Gupta M, Nicholas TJ, Brown J, Baird L, et al De novo structural mutation rates and gamete-of-origin biases revealed through genome sequencing of 2,396 families. Am J Hum Genet. 2021;108(4):597–607. doi:10.1016/j.ajhg.2021.02.012. 33675682 PMC8059337

[iyad161-B5] Benson G . Tandem repeats finder: a program to analyze DNA sequences. Nucleic Acids Res. 1999;27(2):573–580. doi:10.1093/nar/27.2.573. 9862982 PMC148217

[iyad161-B6] Bertolotti AC, Layer RM, Gundappa MK, Gallagher MD, Pehlivanoglu E, Nome T, Robledo D, Kent MP, Røsæg LL, Holen MM, et al The structural variation landscape in 492 Atlantic salmon genomes. Nat Commun. 2020;11(1):5176. doi:10.1038/s41467-020-18972-x 33056985 PMC7560756

[iyad161-B7] Boussaha M, Esquerré D, Barbieri J, Djari A, Pinton A, Letaief R, Salin G, Escudié F, Roulet A, Fritz S, et al Genome-wide study of structural variants in bovine Holstein, Montbéliarde and Normande dairy breeds. PLoS One 2015;10(8):8. doi:10.1371/journal.pone.0135931. PMC4552564 26317361

[iyad161-B8] Broad Institute . Picard tools. 2021. http://broadinstitute.github.io/picard/.

[iyad161-B9] Browning BL, Browning SR. Genotype imputation with millions of reference samples. Am J Hum Genet. 2016;98(1):116–126. doi:10.1016/j.ajhg.2015.11.020. 26748515 PMC4716681

[iyad161-B10] Cao X, Zhang Y, Payer LM, Lords H, Steranka JP, Burns KH, Xing J. Polymorphic mobile element insertions contribute to gene expression and alternative splicing in human tissues. Genome Biol. 2020;21(1):185. doi:10.1186/s13059-020-02101-4. 32718348 PMC7385971

[iyad161-B11] Chaisson MJP, Sanders AD, Zhao X, Malhotra A, Porubsky D, Rausch T, Gardner EJ, Rodriguez OL, Guo L, Collins RL, et al Multi-platform discovery of haplotype-resolved structural variation in human genomes. Nat Commun. 2019;10(1):1784. doi:10.1038/s41467-018-08148-z. 30992455 PMC6467913

[iyad161-B12] Chen L, Chamberlain AJ, Reich CM, Daetwyler HD, Hayes BJ. Detection and validation of structural variations in bovine whole-genome sequence data. Genet Sel Evol. 2017;49(1):31. doi:10.1186/s12711-017-0286-5 28257629 PMC5336645

[iyad161-B13] Chen S, Zhou Y, Chen Y, Gu J. fastp: an ultra-fast all-in-one FASTQ preprocessor. *Bioinformatics*. 2018;34(17):884–890. doi:10.1093/bioinformatics/bty560 30423086 PMC6129281

[iyad161-B14] Chiang C, Layer RM, Faust GG, Lindberg MR, Rose DB, Garrison EP, Marth GT, Quinlan AR, Hall IM. Speedseq: ultra-fast personal genome analysis and interpretation. Nat Methods. 2015;12(10):966–968. doi:10.1038/nmeth.3505. 26258291 PMC4589466

[iyad161-B15] Chiang C, Scott AJ, Davis JR, Tsang EK, Li X, Kim Y, Hadzic T, Damani FN, Ganel L, Montgomery SB, et al The impact of structural variation on human gene expression. Nat Genet. 2017;49(5):692–699. doi:10.1038/ng.3834. 28369037 PMC5406250

[iyad161-B16] Collins RL, Brand H, Karczewski KJ, Zhao X, Alföldi J, Francioli LC, Khera AV, Lowther C, Gauthier LD, Wang H, et al A structural variation reference for medical and population genetics. Nature 2020;581(7809):444–451. doi:10.1038/s41586-020-2287-8. 32461652 PMC7334194

[iyad161-B17] Cotto KC, Feng YY, Ramu A, Richters M, Freshour SL, Skidmore ZL, Xia H, McMichael JF, Kunisaki J, Campbell KM, et al Integrated analysis of genomic and transcriptomic data for the discovery of splice-associated variants in cancer. Nat Commun. 2023;14(1):1589. doi:10.1038/s41467-023-37266-6. 36949070 PMC10033906

[iyad161-B18] Danecek P, Bonfield JK, Liddle J, Marshall J, Ohan V, Pollard MO, Whitwham A, Keane T, McCarthy SA, Davies RM. Twelve years of SAMtools and BCFtools. Gigascience 2021;10(2):2. doi:10.1093/gigascience/giab008. PMC7931819 33590861

[iyad161-B19] Delaneau O, Ongen H, Brown AA, Fort A, Panousis NI, Dermitzakis ET. A complete tool set for molecular QTL discovery and analysis. Nat Commun. 2017;8(1):15452. doi:10.1038/ncomms15452. 28516912 PMC5454369

[iyad161-B20] Depristo MA, Banks E, Poplin R, Garimella KV, Maguire JR, Hartl C, Philippakis AA, Del Angel G, Rivas MA, Hanna M, et al A framework for variation discovery and genotyping using next-generation DNA sequencing data. Nat Genet. 2011;43(5):491–501. doi:10.1038/ng.806. 21478889 PMC3083463

[iyad161-B21] Dobin A, Davis CA, Schlesinger F, Drenkow J, Zaleski C, Jha S, Batut P, Chaisson M, Gingeras TR. STAR: ultrafast universal RNA-seq aligner. Bioinformatics 2013;29(1):15–21. doi:10.1093/bioinformatics/bts635. 23104886 PMC3530905

[iyad161-B22] Ebert P, Audano PA, Zhu Q, Rodriguez-Martin B, Porubsky D, Bonder MJ, Sulovari A, Ebler J, Zhou W, Mari RS, et al Haplotype-resolved diverse human genomes and integrated analysis of structural variation. Science 2021;372(6537):6537. doi:10.1126/science.abf7117. PMC8026704 33632895

[iyad161-B23] Ellegren H . Heterogeneous mutation processes in human microsatellite DNA sequences. Nat Genet. 2000;24(4):400–402. doi:10.1038/74249 10.1038/74249. 10742106

[iyad161-B24] Escaramís G, Docampo E, Rabionet R. A decade of structural variants: description, history and methods to detect structural variation. Brief Funct Genomics. 2015;14(5):305–314. doi:10.1093/bfgp/elv014. 25877305

[iyad161-B25] Faust GG, Hall IM. 2014. SAMBLASTER: fast duplicate marking and structural variant read extraction. Bioinformatics 30(17):2503–2505. doi:10.1093/bioinformatics/btu314. doi:10.1093/bioinformatics/btu314. 24812344 PMC4147885

[iyad161-B26] Fotsing SF, Margoliash J, Wang C, Saini S, Yanicky R, Shleizer-Burko S, Goren A, Gymrek M. The impact of short tandem repeat variation on gene expression. Nat Genet. 2019;51(11):1652–1659. doi:10.1038/s41588-019-0521-9. 31676866 PMC6917484

[iyad161-B27] Gamazon ER, Segrè AV, Van De Bunt M, Wen X, Xi HS, Hormozdiari F, Ongen H, Konkashbaev A, Derks EM, Aguet F, et al Using an atlas of gene regulation across 44 human tissues to inform complex disease- and trait-associated variation. Nat Genet. 2018;50(7):956–967. doi:10.1038/s41588-018-0154-4. 29955180 PMC6248311

[iyad161-B28] Garrido-Martín D, Borsari B, Calvo M, Reverter F, Guigó R. Identification and analysis of splicing quantitative trait loci across multiple tissues in the human genome. Nat Commun. 2021;12(1):727. doi:10.1038/s41467-020-20578-2. 33526779 PMC7851174

[iyad161-B29] Grapotte M, Saraswat M, Bessière C, Menichelli C, Ramilowski JA, Severin J, Hayashizaki Y, Itoh M, Tagami M, Murata M, et al Discovery of widespread transcription initiation at microsatellites predictable by sequence-based deep neural network. Nat Commun. 2021;12(1):3297. doi:10.1038/s41467-021-23143-7. 34078885 PMC8172540

[iyad161-B30] The GTEx Consortium . The GTEx Consortium atlas of genetic regulatory effects across human tissues. Science 2020;369(6509):1318–1330. doi:10.1126/science.aaz1776. 32913098 PMC7737656

[iyad161-B31] Gustavsson EK, Zhang D, Reynolds RH, Garcia-Ruiz S, Ryten M. ggtranscript: an R package for the visualization and interpretation of transcript isoforms using ggplot2. Bioinformatics. 2022;38(15):3844–3846. doi:10.1093/bioinformatics/btac409 10.1093/bioinformatics/btac409. 35751589 PMC9344834

[iyad161-B32] Gymrek M . A genomic view of short tandem repeats. Curr Opin Genet Dev. 2017;44(1):9–16. doi:10.1016/j.gde.2017.01.012 28213161

[iyad161-B33] Hamanaka K, Yamauchi D, Koshimizu E, Watase K, Mogushi K, Ishikawa K, Mizusawa H, Tsuchida N, Uchiyama Y, Fujita A, et al Genome-wide identification of tandem repeats associated with splicing variation across 49 tissues in humans. Genome Res. 2023;33(3):435–447. doi:10.1101/gr.277335.122. 37307504 PMC10078293

[iyad161-B34] Ho SS, Urban AE, Mills RE. Structural variation in the sequencing era. Nat Rev Genet. 2020;21(3):171–189. doi:10.1038/s41576-019-0180-9. 31729472 PMC7402362

[iyad161-B35] Hoffman GE, Schadt EE. variancePartition: interpreting drivers of variation in complex gene expression studies. BMC Bioinformatics 2016;17(1):483. doi:10.1186/s12859-016-1323-z. 27884101 PMC5123296

[iyad161-B36] Ihara N, Takasuga A, Mizoshita K, Takeda H, Sugimoto M, Mizoguchi Y, Hirano T, Itoh T, Watanabe T, Reed KM, et al A comprehensive genetic map of the cattle genome based on 3802 microsatellites. Genome Res. 2004;14(10a):1987–1998. doi:10.1101/gr.2741704. 15466297 PMC524423

[iyad161-B37] Jakubosky D, D’Antonio M, Bonder MJ, Smail C, Donovan MKR, Young Greenwald WW, Bonder Marc J., Cai Na, Carcamo-Orive Ivan, Matsui H, et al Properties of structural variants and short tandem repeats associated with gene expression and complex traits. Nat Commun. 2020;11(1):2927. doi:10.1038/s41467-020-16482-4. 32522982 PMC7286898

[iyad161-B38] Kadri NK, Sahana G, Charlier C, Iso-Touru T, Guldbrandtsen B, Karim L, Nielsen US, Panitz F, Aamand GP, Schulman Net al A 660-Kb deletion with antagonistic effects on fertility and milk production segregates at high frequency in Nordic Red cattle: additional evidence for the common occurrence of balancing selection in livestock. PLoS Genet. 2014;10(1):e1004049. 10.1371/journal.pgen.1004049 24391517 PMC3879169

[iyad161-B39] Kadri NK, Marie Mapel X, Pausch H. The intronic branch point sequence is under strong evolutionary constraint in the bovine and human genome. Commun Biol. 2021;4(1):1206. doi:10.1038/s42003-021-02725-7. 34675361 PMC8531310

[iyad161-B40] Kommadath A , Grant JR, Krivushin K, Butty AM, Baes CF, Carthy TR, Berry DP, Stothard P. A large interactive visual database of copy number variants discovered in taurine cattle. Gigascience. 2019;8(6):giz073. 10.1093/gigascience/giz073 31241156 PMC6593363

[iyad161-B41] Layer RM, Chiang C, Quinlan AR, Hall IM. 2014. LUMPY: a probabilistic framework for structural variant discovery. Genome Biol. 15(6):R84. doi:10.1186/gb-2014-15-6-r84. doi:10.1186/gb-2014-15-6-r84. 24970577 PMC4197822

[iyad161-B42] Lee Y-L, Bosse M, Takeda H, Moreira GCM, Karim L, Druet T, Oget-Ebrad C, Coppieters W, Veerkamp RF, Groenen MAM, et al High-resolution structural variants catalogue in a large-scale whole genome sequenced bovine family cohort data. BMC Genomics 2023;24(1):225. doi:10.1186/s12864-023-09259-8. 37127590 PMC10152703

[iyad161-B43] Lee YL, Takeda H, Moreira GCM, Karim L, Mullaart E, Coppieters W, Appeltant R, Veerkamp RF, Groenen MAM, Georges M, et al A 12kb multi-allelic copy number variation encompassing a GC gene enhancer is associated with mastitis resistance in dairy cattle. PLoS Genet. 2021;17(7):e1009331. doi:10.1371/journal.pgen.1009331. 34288907 PMC8328317

[iyad161-B44] Leonard AS, Crysnanto D, Fang ZH, Heaton MP, Vander Ley BL, Herrera C, Bollwein H, Bickhart DM, Kuhn KL, Smith TPL, et al Structural variant-based pangenome construction has low sensitivity to variability of haplotype-resolved bovine assemblies. Nat Commun. 2022;13(1):3012. doi:10.1038/s41467-022-30680-2. 35641504 PMC9156671

[iyad161-B45] Leonard AS, Mapel XM, Pausch H. 2023. Pangenome genotyped structural variation improves molecular phenotype mapping in cattle. bioRxiv. doi:10.1101/2023.06.21.545879.

[iyad161-B46] Li H . 2013. Aligning sequence reads, clone sequences and assembly contigs with BWA-MEM. arXiv:1303.3997. 1–3. doi:10.48550/arXiv.1303.3997.

[iyad161-B47] Li YI, Knowles DA, Humphrey J, Barbeira AN, Dickinson SP, Im HK, Pritchard JK. Annotation-free quantification of RNA splicing using LeafCutter. Nat Genet. 2018;50(1):151–158. doi:10.1038/s41588-017-0004-9. 29229983 PMC5742080

[iyad161-B48] Liao Y, Smyth GK, Shi W. featureCounts: an efficient general purpose program for assigning sequence reads to genomic features. Bioinformatics 2014;30(7):923–930. doi:10.1093/bioinformatics/btt656. 24227677

[iyad161-B49] Littlejohn MD, Tiplady K, Fink TA, Lehnert K, Lopdell T, Johnson T. Sequence-based association analysis reveals an MGST1 eQTL with pleiotropic effects on bovine milk composition. Sci Rep. 2016;6(1):25376. doi:10.1038/srep25376. 27146958 PMC4857175

[iyad161-B50] Liu S, Gao Y, Canela-Xandri O, Wang S, Yu Y, Cai W, Li B, Xiang R, Chamberlain AJ, Pairo-Castineira E, et al A multi-tissue atlas of regulatory variants in cattle. Nat Genet. 2022;54(9):1438–1447. doi:10.1038/s41588-022-01153-5. 35953587 PMC7613894

[iyad161-B51] Lloret-Villas A, Bhati M, Kadri NK, Fries R, Pausch H. Investigating the impact of reference assembly choice on genomic analyses in a cattle breed. BMC Genomics 2021;22(1):363. doi:10.1186/s12864-021-07554-w. 34011274 PMC8132449

[iyad161-B52] Lopdell TJ, Tiplady K, Struchalin M, Johnson TJJ, Keehan M, Sherlock R, Couldrey C, Davis SR, Snell RG, Spelman RJ, et al DNA and RNA-sequence based GWAS highlights membrane-transport genes as key modulators of milk lactose content. BMC Genomics 2017;18(1):968. doi:10.1186/s12864-017-4320-3. 29246110 PMC5731188

[iyad161-B53] Machugh DE, Shriver MD, Loftus RT, Cunningham P, Bradley DG. Microsatellite DNA variation and the evolution, domestication and phylogeography of taurine and zebu cattle (*Bos taurus* and *Bos indicus*). Genetics 1994;146(3):1071–1086. doi:10.1098/rspb.1994.0044. PMC1208036 9215909

[iyad161-B54] Mahmoud M, Gobet N, Cruz-Dávalos DI, Mounier N, Dessimoz C, Sedlazeck FJ. Structural variant calling: the long and the short of it. Genome Biol. 2019;20(1):246. doi:10.1186/s13059-019-1828-7 31747936 PMC6868818

[iyad161-B55] Mapel XM, Kadri NK, Leonard AS, He Q, Lloret-Villas A, Bhati M, Hiltpold M, Pausch H. 2023. Molecular quantitative trait loci in reproductive tissues impact male fertility in a large mammal. bioRxiv. doi:10.1101/2023.06.29.547066. PMC10803364 38253538

[iyad161-B56] McCaw ZR, Lane JM, Saxena R, Redline S, Lin X. Operating characteristics of the rank-based inverse normal transformation for quantitative trait analysis in genome-wide association studies. Biometrics 2020;76(4):1262–1272. doi:10.1111/biom.13214. 31883270 PMC8643141

[iyad161-B57] McClure M, Sonstegard T, Wiggans G, Van Tassell CP. Imputation of microsatellite alleles from dense SNP genotypes for parental verification. Front Genet. 2012;3(9):140. doi:10.3389/fgene.2012.00140 22912645 PMC3418578

[iyad161-B58] McLaren W, Gil L, Hunt SE, Riat HS, Ritchie GRS, Thormann A, Flicek P, Cunningham F. The Ensembl Variant Effect Predictor. Genome Biol. 2016;17(1):122. doi:10.1186/s13059-016-0974-4. 27268795 PMC4893825

[iyad161-B59] Mesbah-Uddin M, Guldbrandtsen B, Iso-Touru T, Vilkki J, De Koning DJ, Boichard D, Lund MS, Sahana G. Genome-wide mapping of large deletions and their population-genetic properties in dairy cattle. DNA Res. 2018;25(1):49–59. doi:10.1093/dnares/dsx037. 28985340 PMC5824824

[iyad161-B60] Pedersen TL . 2023. patchwork: the vcomposer of plots. https://github.com/thomasp85/patchwork.

[iyad161-B61] Pedersen BS, Layer R, Quinlan AR. 2020. smoove: structural-variant calling and genotyping with existing tools. https://github.com/brentp/smoove.

[iyad161-B62] Pedersen BS, Quinlan AR. Duphold: scalable, depth-based annotation and curation of high-confidence structural variant calls. Gigascience 2019;8(4):4. doi:10.1093/gigascience/giz040. PMC6479422 31222198

[iyad161-B63] Purcell S, Neale B, Todd-Brown K, Thomas L, Ferreira MAR, Bender D, Maller J, Sklar P, de Bakker PIW, Daly MJ, et al PLINK: a tool set for whole-genome association and population-based linkage analyses. Am J Hum Genet. 2007;81(3):559–575. doi:10.1086/519795. 17701901 PMC1950838

[iyad161-B64] Quinlan AR, Hall IM. BEDTools: a flexible suite of utilities for comparing genomic features. Bioinformatics 2010;26(6):841–842. doi:10.1093/bioinformatics/btq033. 20110278 PMC2832824

[iyad161-B65] Rafehi H, Szmulewicz DJ, Bennett MF, Sobreira NLM, Pope K, Smith KR, Gillies G, Diakumis P, Dolzhenko E, Eberle MA, et al Bioinformatics-based identification of expanded repeats: a non-reference intronic pentamer expansion in RFC1 causes CANVAS. Am J Hum Genet. 2019;105(1):151–165. doi:10.1016/j.ajhg.2019.05.016. 31230722 PMC6612533

[iyad161-B66] Saini S, Mitra I, Mousavi N, Fotsing SF, Gymrek M. A reference haplotype panel for genome-wide imputation of short tandem repeats. Nat Commun. 2018;9(1):4397. doi:10.1038/s41467-018-06694-0. 30353011 PMC6199332

[iyad161-B67] Scott AJ, Chiang C, Hall IM. Structural variants are a major source of gene expression differences in humans and often affect multiple nearby genes. Genome Res. 2021;31(12):2249–2258. doi:10.1101/gr.275488.121. 34544830 PMC8647827

[iyad161-B68] Shaul O . How introns enhance gene expression. Int J Biochem Cell Biol. 2017;91(1):145–155. doi:10.1016/j.biocel.2017.06.016 28673892

[iyad161-B69] Sinnott-Armstrong N, Naqvi S, Rivas M, Pritchard JK. GWAS of three molecular traits highlights core genes and pathways alongside a highly polygenic background. Elife 2021;10(1):e58615. doi:10.7554/eLife.58615 33587031 PMC7884075

[iyad161-B70] Stegle O, Parts L, Piipari M, Winn J, Durbin R. Using probabilistic estimation of expression residuals (PEER) to obtain increased power and interpretability of gene expression analyses. Nat Protoc. 2012;7(3):500–507. doi:10.1038/nprot.2011.457. 22343431 PMC3398141

[iyad161-B71] Sudmant PH, Rausch T, Gardner EJ, Handsaker RE, Abyzov A, Huddleston J, Zhang Y, Ye K, Jun G, Fritz MHY, et al An integrated map of structural variation in 2,504 human genomes. Nature 2015;526(7571):75–81. doi:10.1038/nature15394. 26432246 PMC4617611

[iyad161-B72] Sun JX, Helgason A, Masson G, Ebenesersdóttir SS, Li H, Mallick S, Gnerre S, Patterson N, Kong A, Reich D, et al A direct characterization of human mutation based on microsatellites. Nat Genet. 2012;44(10):1161–1165. doi:10.1038/ng.2398. 22922873 PMC3459271

[iyad161-B73] Talenti A, Powell J, Hemmink JD, Cook EAJ, Wragg D, Jayaraman S, Paxton E, Ezeasor C, Obishakin ET, Agusi ER, et al A cattle graph genome incorporating global breed diversity. Nat Commun. 2022;13(1):910. doi:10.1038/s41467-022-28605-0. 35177600 PMC8854726

[iyad161-B74] van de Geijn B, McVicker G, Gilad Y, Pritchard JK. 2015. WASP: allele-specific software for robust molecular quantitative trait locus discovery. Nat Methods. 12(11):1061–1063. doi:10.1038/nmeth.3582. doi:10.1038/nmeth.3582. 26366987 PMC4626402

[iyad161-B75] Van De Goor LHP, Panneman H, Van Haeringen WA. A proposal for standardization in forensic bovine DNA typing: allele nomenclature of 16 cattle-specific short tandem repeat loci. Anim Genet. 2009;40(5):630–636. doi:10.1111/j.1365-2052.2009.01891.x. 19397508

[iyad161-B76] Vialle RA, de Paiva Lopes K, Bennett DA, Crary JF, Raj T. Integrating whole-genome sequencing with multi-omic data reveals the impact of structural variants on gene regulation in the human brain. Nat Neurosci. 2022;25(4):504–514. doi:10.1038/s41593-022-01031-7. 35288716 PMC9245608

[iyad161-B77] Visscher PM, Brown MA, McCarthy MI, Yang J. Five years of GWAS discovery. Am J Hum Genet. 2012;90(1):7–24. doi:10.1016/j.ajhg.2011.11.029. 22243964 PMC3257326

[iyad161-B78] Wang K, Li M, Hadley D, Liu R, Glessner J, Grant SFA, Hakonarson H, Bucan M. PennCNV: an integrated hidden Markov model designed for high-resolution copy number variation detection in whole-genome SNP genotyping data. Genome Res. 2007;17(11):1665–1674. doi:10.1101/gr.6861907. 17921354 PMC2045149

[iyad161-B79] Willems T, Gymrek M, Highnam G, Mittelman D, Erlich Y. The landscape of human STR variation. Genome Res. 2014;24(11):1894–1904. doi:10.1101/gr.177774.114. 25135957 PMC4216929

[iyad161-B80] Willems T, Zielinski D, Yuan J, Gordon A, Gymrek M, Erlich Y. Genome-wide profiling of heritable and de novo STR variations. Nat Methods. 2017;14(6):590–592. doi:10.1038/nmeth.4267. 28436466 PMC5482724

[iyad161-B81] Wu Z, Gong H, Zhang M, Tong X, Ai H, Xiao S, Perez-Enciso M, Yang B, Huang L. A worldwide map of swine short tandem repeats and their associations with evolutionary and environmental adaptations. Genet Select Evol. 2021;53(1):39. doi:10.1186/s12711-021-00631-4. PMC8063339 33892623

[iyad161-B82] Xiang R, Fang L, Liu S, Macleod IM, Liu Z, Breen EJ, Gao Y, Liu GE, Tenesa A, CattleGTEx Consortium, et al 2022. Gene expression and RNA splicing explain large proportions of the heritability for complex traits in cattle. bioRxiv. 10.1101/2022.05.30.494093.

[iyad161-B83] Xu L, Haasl RJ, Sun J, Zhou Y, Bickhart DM, Li J, Song J, Sonstegard TS, Van Tassell CP, Lewin HA, et al Systematic profiling of short tandem repeats in the cattle genome. Genome Biol Evol. 2017;9(1):20–31. doi:10.1093/gbe/evw256. 28172841 PMC5381564

[iyad161-B84] Yang J, Ferreira T, Morris AP, Medland SE, Madden PAF, Heath AC, Martin NG, Montgomery GW, Weedon MN, Loos RJ, et al Conditional and joint multiple-SNP analysis of GWAS summary statistics identifies additional variants influencing complex traits. Nat Genet. 2013;44(4):369–375. doi:10.1038/ng.2213. PMC3593158 22426310

